# Synthesis of methylenebisamides using CC- or DCMT-activated DMSO

**DOI:** 10.3762/bjoc.4.51

**Published:** 2008-12-15

**Authors:** Qiang Wang, Lili Sun, Yu Jiang, Chunbao Li

**Affiliations:** 1 Department of Chemistry, College of Science, Tianjin University, Tianjin 300072, China; 2 State Key Laboratory Base of Novel Functional Materials and Preparation Science at Ningbo, Faculty of Materials Science and Chemical Engineering, Ningbo University, Ningbo 315211, China.

**Keywords:** amides, condensation, DMSO, methylenebisamides, 2,4,6-trichloro[1,3,5]triazine

## Abstract

Bisamides are key fragments for the introduction of *gem*-diaminoalkyl residues into retroinverso pseudopeptide derivatives and in the synthesis of peptidomimetic compounds. The literature methods for these types of compounds have certain drawbacks. In particular, when amides react with electrophile-activated DMSO, the yields are rather low. We have found new electrophiles, 2,4,6-trichloro[1,3,5]triazine (CC) and 2,4-dichloro-6-methoxy[1,3,5]triazine (DCMT), which activate DMSO in the presence of amides to yield methylenebisamides in good to fair yields. The amides can be aromatic or aliphatic. The operation is simple and the reagents are inexpensive.

## Introduction

Sulfoxides are activated by electrophiles to produce reactive sulfonium salts. These electrophiles include DCC [1–2], acetic anhydride [3], trifluoroacetic anhydride [4], acetyl chloride [5], phosphorus pentoxide [6], polyphosphoric acid [7], sulfuric acid and sulfur trioxide [6] etc. A few reactions such as the Pummerer reaction [8] and Swern oxidation [9] can be performed via the reactive sulfonium salts formed from DMSO and electrophiles.

The reactions of amides with DMSO in presence of electrophiles (sulfur trioxide, acetic anhydride, phosphorus pentoxide) give *N*,*N*′-methylenebisamides and *N*-acylsulfilimines, but rather low yields of *N*,*N*′-methylenebisamides (20%) [6]. From our previous research on the chlorination [10] and etherification [11] of benzyl alcohols and from other references [12–14], we believe the reaction between 2,4,6-trichloro[1,3,5]triazine (cyanogen chloride, or CC) and DMSO produces a reactive sulfonium salt intermediate. Therefore, it was of interest to study the reaction of nucleophilic reagents such as amides, alcohols, and phenols with DMSO activated by CC.

The amide moiety is an important constituent of many biologically significant compounds. Bisamides are of considerable interest in the synthesis of peptidomimetic compounds [15]. In particular, bisamides are key fragments for the introduction of *gem*-diaminoalkyl residues in retro-inverso pseudopeptide derivatives [16] by treating the corresponding amide with iodobenzene bistrifluoroacetate [17]. *N*,*N*′-Methylenebisamides are usually prepared by the reactions of amides with aldehydes [18–20], hexamethylenetetramine [21] or activated DMSO [6], or by the reaction of nitriles with formaldehyde [22] or activated sulfoxides [23]. However, each method has certain limitations with regards to scope and reaction conditions, for example, longer reaction time [20], lower yield [6], purification problems [21,23] and drastic reaction conditions [19,22].

In this paper, we report the reaction of amides with DMSO activated by CC or 2,4-dichloro-6-methoxy[1,3,5]triazine (DCMT) [24].

## Results and Discussion

**Scheme 1:**
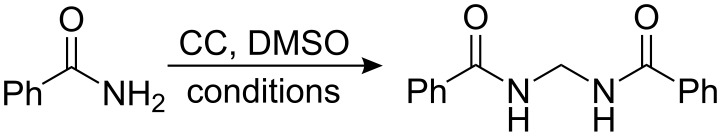
Synthesis of *N*,*N*′-methylenedibenzamide using CC-activated DMSO.

Initially, we chose CC as the activation reagent and benzamide as a model substrate to optimize the reaction conditions (Scheme 1, Table 1). No product was observed when the reaction was carried out in CH_3_CN (Table 1, entry 1). The reaction proceeded in CHCl_3_, DMSO, EtOAc and toluene (Table 1, entries 2–5), but considerable amounts of undesired products were formed in CHCl_3_, DMSO and EtOAc (Table 1, entries 2–4). Better results were obtained when the reactions were performed in toluene (Table 1, entry 5). Encouraged by these results, we studied the effects of temperature on the reaction in toluene. Elevating the temperature to 70 °C resulted in an improved reaction rate. As for the influence of CC and DMSO dosage on the reaction, it was found that decreasing the amount of CC to 0.9 equiv resulted in reduced yield, while increasing the amount to 1.5 equiv did not make the reaction system complex and the yield was not notably different. Excess amount of DMSO (7.0 equiv) was used partially because of its ability to dissolve the amides.

**Table 1:** Optimization studies for the synthesis of *N*,*N*′-methylenedibenzamide using CC-activated DMSO^a^.

Entry	Solvent	Temp (°C)	t (h)	Yield (%)^b^

1	CH_3_CN	RT	24	0
2	CHCl_3_	RT	4	10^c^
3	DMSO	RT	24	26
4	EtOAc	RT	4	30
5	Toluene	RT	4	70
6	Toluene	RT to 70	2	71^d^

^a^1.0 equiv amide, 1.2 equiv CC, 7.0 equiv dry DMSO, dry toluene (8.0 mL). ^b^Isolated yield; ^c^*S*,*S*-Dimethyl-*N*-benzoylsulfilimine (40% yield) as major product; ^d^Stirred for 30 min at RT, then 70 °C for 1.5 h.

**Scheme 2:**
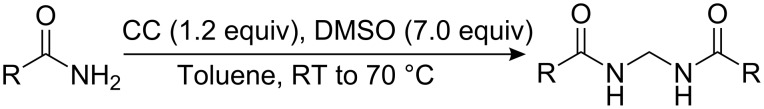
Synthesis of *N*,*N*′-methylenebisamide using CC-activated DMSO.

After the optimization of the reaction conditions, various substrates were subjected to the conditions (Scheme 2, Table 2). The electronic effect of the substituents on aromatic rings was observed. For example, a strong electron-donating or electron-withdrawing group on the aromatic ring resulted in decreased reaction yields (Table 2, entries 5, 8), while a substituent on the para-position or meta-position led to a moderate yield (Table 2, entries 4, 7). The effect of substituents on the reaction rate was not remarkable. However, the method was not efficient for the aliphatic amides: considerable amounts of by-products were formed, probably due to the greater nucleophilicity of the aliphatic amides.

**Table 2:** Scope of the synthesis of *N*,*N*′-methylenebisamide using CC-activated DMSO^a^.

Entry	R	t (h)	Yield (%)^b^

1	Ph	2	71
2	4-CH_3_C_6_H_4_	4	86
3	4-MeOC_6_H_4_	2	72
4	2-MeOC_6_H_4_	1.5	67
5	3,5-(MeO)_2_C_6_H_3_	4	30
6	4-ClC_6_H_4_	5	74
7	3-ClC_6_H_4_	4	60
8	4-NO_2_C_6_H_4_	4	20
9	PhCH=CH	3	50
10	PhCH_2_	–	–^c^
11	PhOCH_2_	–	–^c^
12	(CH_3_)_3_C	–	–^c^
13	CH_3_(CH_2_)_5_CH_2_	–	–^c^

^a^1.0 equiv amide, 1.2 equiv CC, 7.0 equiv dry DMSO, dry toluene (8.0 mL). ^b^Isolated yield; ^c^Complicated products.

**Scheme 3:**
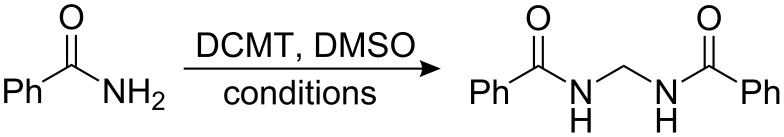
Synthesis of *N*,*N*′-methylenedibenzamide using DCMT-activated DMSO.

**Scheme 4:**
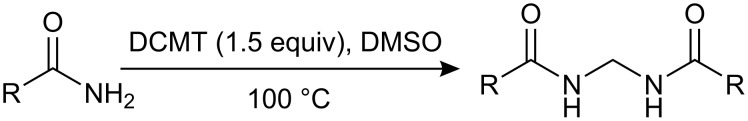
Synthesis of *N*,*N*′-methylenebisamide using DCMT-activated DMSO.

In order to improve the yield of aliphatic amides, less active DCMT was used as activating reagent instead of CC. We also optimized the reaction conditions using benzamide as a benchmark (Scheme 3, Table 3). The reaction did not proceed when low boiling solvents such as CH_3_CN, CHCl_3_ and ClCH_2_CH_2_Cl were used (Table 3, entries 1–3). It was performed efficiently in high boiling solvents (1,4-dioxane, xylene and DMSO) (Table 3, entries 4–6). The results indicate that the effect of temperature on the reaction is remarkable. The electronic effect of the substituents on aromatic rings was similar to the results when CC was used as an activating reagent (Scheme 4, Table 4, entries 5, 8). The system of DCMT/DMSO was efficient for aliphatic amides (Scheme 4, Table 4, entries 10–13).

**Table 3:** Optimization studies for the synthesis of *N*,*N*′-methylenedibenzamide using DCMT-activated DMSO^a^.

Entry	Solvent	Temp (°C)	t (h)	Yield (%)^a^

1	CH_3_CN	Reflux	24	0
2	CHCl_3_	Reflux	24	0
3	1,2-Dichloroethane	Reflux	24	0
4	Dioxane	100	3	75
5	Xylene	100	3	70
6	DMSO	100	1	75

^a^1.0 equiv amide, 1.5 equiv DCMT, dry DMSO (4.0 mL), 100 °C. ^b ^Isolated yield.

**Table 4:** Scope of the synthesis of *N*,*N*′-methylenebisamide using DCMT-activated DMSO^a.^

Entry	R	t (h)	Yield (%)^b^

1	Ph	1	75
2	4-CH_3_C_6_H_4_	1	77
3	4-MeOC_6_H_4_	1	70
4	2-MeOC_6_H_4_	0.5	52
5	3,5-(MeO)_2_C_6_H_3_	3^c^	50
6	4-ClC_6_H_4_	1	88
7	3-ClC_6_H_4_	1.5	54
8	4-NO_2_C_6_H_4_	3	28
9	PhCH=CH	1	55
10	PhCH_2_	4	45
11	PhOCH_2_	2	62
12	(CH_3_)_3_C	1	60
13	CH_3_(CH_2_)_5_CH_2_	3	20^d^

^a^1.0 equiv amide, 1.5 equiv DCMT, dry DMSO (4.0 mL), 100 °C. ^b ^Isolated yield; ^c^The reaction performed at 70 °C; ^d^*N*-(Methylthiomethyl)octanamide (40% yield) as major product.

**Scheme 5:**
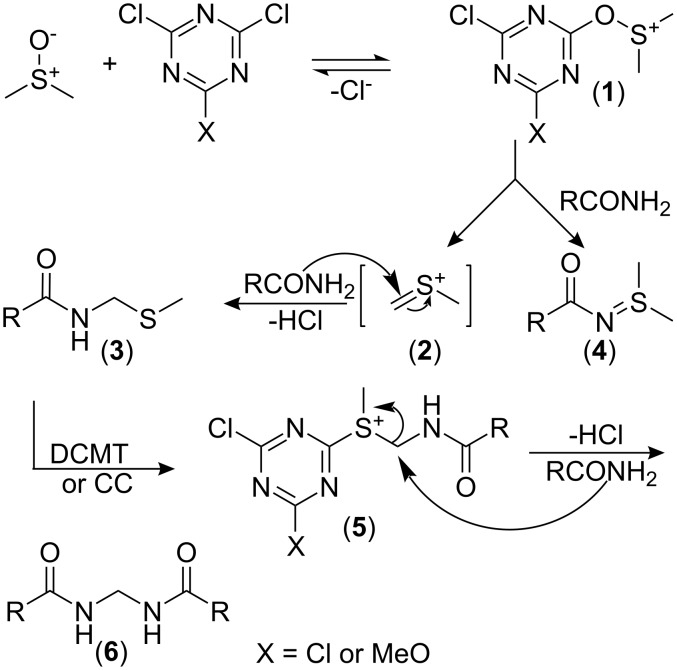
Plausible reaction mechanism of amide with CC- or DCMT-activated DMSO.

Based on these experiments, a possible mechanism [6,23,25] is shown in Scheme 5. Intermediate **1** reacted with amides by two pathways. When the reaction of benzamide and CC-activated DMSO was carried out in chloroform, *S*,*S*-dimethyl-*N*-benzoylsulfilimine (**4**) was isolated as a major product, most probably formed by the attack of the amide on the sulfonium ion **1**. When the reaction is carried out in toluene, intermediate **1** decomposes into intermediate **2**. Thioether **3** is formed by the addition of the amide to intermediate **2**. *N*-(Methylthiomethyl)octanamide (40% yield) was isolated when octanamide was treated with DCMT in DMSO. Similarly, *N*-(1-(methylthio)-2-oxo-2-phenylethyl)benzamide (30% yield) was isolated when benzamide reacted with DCMT and 2-(methylsulfinyl)-1-phenylethanone. Thioether **3** is a good nucleophile and capable to substitute the chloride of CC or DCMT to generate sulfonium salt **5**. The amide substitutes the thioether of **5** to form methylenebisamides **6**.

## Conclusion

In conclusion, we have developed a simple and efficient procedure to produce methylenebisamides in good yield via the reaction of amides with CC- or DCMT-activated DMSO. The procedure reported herein is operationally simple, and requires inexpensive and commercially available reagents. A plausible mechanism of the reaction which involves two sulfonium salt intermediates was proposed and supported by the experiments.

## Experimental

All chemicals were obtained from commercial sources or prepared according to standard methods [24]. All chemicals and solvents used in the reactions were dried by standard procedures prior to use. IR spectra were recorded on a Bio-Rad Exalibur FTS3000 spectrometer. The ^1^H NMR (500 MHz) and ^13^C NMR (125 MHz) were recorded on a Varian Oxford 500 spectrometer. Chemical shifts (δ) are reported relative to TMS (^1^H) in DMSO-*d*_6_ or CDCl_3_ (^13^C). Mass spectra were obtained using an LCQ Advantage MAX ion trap mass spectrometer equipped with electrospray ionization (ESI) ion source or a Thermo Finnigan TRACE-DSQ spectrometer. Elemental analyses for C, H and N were performed on a Yanaco CHNCORNER MF-3 elemental analyzer, and the analytical results were within ±0.4% of the theoretical values.

### Typical experimental procedure

#### *N*,*N*′-Methylenedibenzamide (Table 2, entry 1)

A mixture of amide (121.4 mg, 1.0 mmol, 1 equiv), CC (222 mg, 1.2 mmol, 1.2 equiv) and dry DMSO (0.5 mL, 7.0 mmol, 7.0 equiv) in dry toluene (8.0 mL) was stirred for 30 min at room temperature. The reaction temperature was then kept at 70 °C for 1.5 h until completion. The mixture was neutralized with saturated aqueous NaHCO_3_ (20 mL), then extracted with EtOAc (3 × 20 mL). The extract was washed with brine (4 × 15 mL), dried over anhydrous Na_2_SO_4_. The solvent was concentrated in vacuo to give the crude product, which was further purified by silica gel column chromatography (PE/EA = 1/1) to afford *N*,*N*′-methylenedibenzamide (90.1 mg, 71% yield).

#### *N*,*N*′-Methylenedibenzamide (Table 4, entry 1)

A mixture of amide (121.4 mg, 1.0 mmol, 1 equiv) and DCMT (270 mg, 1.5 mmol, 1.5 equiv) in dry DMSO (4.0 mL) was stirred at 100 °C and monitored by TLC until completion (1.0 h). The working up was similar to Table 2, entry 1 (95.3 mg, 75% yield).

## Supporting Information

File 1 Experimental part

File 2 NMR spectra of new compounds
