# Supplementary material for: Synthesis of methylenebisamides using CC- or DCMT-activated DMSO
Source: Beilstein J Org Chem. 2008 Dec 15;4:51. doi: 10.3762/bjoc.4.51 (PMC2633661; doi:10.3762/bjoc.4.51)
Supplement: File 1 — Experimental part [file Beilstein_J_Org_Chem-04-51-s001.doc]

Supporting information

Synthesis of methylenebisamides using CC- or DCMT-activated DMSO

Qiang Wang^1,2^, Lili Sun^1^, Yu Jiang^1^ and Chunbao Li^1^,*

Address: ^1^Department of Chemistry, College of Science, Tianjin University, Tianjin 300072, China and ^2^State Key Laboratory Base of Novel Functional Materials and Preparation Science at Ningbo, Faculty of Materials Science and Chemical Engineering, Ningbo University, Ningbo 315211, China.

Email: Chunbao Li* - [lichunbao@tju.edu.cn](mailto:lichunbao@tju.edu.cn)

* Corresponding author

**(1) General experimental methods.**

All chemicals were obtained from commercial sources or prepared according to standard methods [1]. All chemicals and solvents used in the reactions were dried by standard procedures prior to use. IR spectra were recorded on a Bio-Rad Exalibur FTS3000 spectrometer. The ^1^H NMR (500 MHz) and ^13^C NMR (125 MHz) were recorded on a Varian Oxford 500 spectrometer. Chemical shifts (δ) are reported relative to TMS (^1^H) in DMSO-*d*_6_ or CDCl_3_ (^13^C). Mass spectra were obtained using an LCQ Advantage MAX ion trap mass spectrometer equipped with electrospray ionization (ESI) ion source or a Thermo Finnigan TRACE-DSQ spectrometer. Elemental analyses for C, H and N were performed on a Yanaco CHNCORNER MF-3 elemental analyzer, and the analytical results were within ±0.4% of the theoretical values.

**(2) Typical experimental procedure:**

***N*,*N'*-Methylenedibenzamide (Table 2, entry 1):**

A mixture of amide (121.4 mg, 1.0 mmol, 1 equiv), CC (222 mg, 1.2 mmol, 1.2 equiv) and dry DMSO (0.5 mL, 7.0 mmol, 7.0 equiv) in dry toluene (8.0 mL) was stirred for 30 min at room temperature. The reaction temperature was then kept at 70 °C for 1.5 h until completion. The mixture was neutralized with saturated aqueous NaHCO_3_ (20 mL), then extracted with EtOAc (3 × 20 mL). The extract was washed with brine (4 × 15 mL), dried over anhydrous Na_2_SO_4_. The solvent was concentrated in vacuo to give the crude product, which was further purified by silica gel column chromatography (PE/EA = 1/1) to afford *N*,*N’*-methylenedibenzamide (90.1 mg, 71% yield).

***N*,*N'*-Methylenedibenzamide (Table 4, entry 1):**

A mixture of amide (121.4 mg, 1.0 mmol, 1 equiv) and DCMT (270 mg, 1.5 mmol, 1.5 equiv) in dry DMSO (4.0 mL) was stirred at 100 °C and monitored by TLC until completion (1.0 h). The work up was similar to the one described for compound Table 2, entry 1 (95.3 mg, 75% yield).

***N*,*N'*-Methylenebis(2-methoxybenzamide) (Table 2, Table 4, entry 4):**

EA/PE = 1/1 as eluant, white solid. ^1^H NMR (500 MHz, CDCl_3_) δ 8.852 (s, 2H), 8.194 (dd, *J*_1_ = 8.0 Hz, *J*_2_ = 2.0 Hz, 2H), 7.463–7.429 (m, 2H), 7.075–7.044 (m, 2H), 6.958 (d, *J* = 8.0 Hz, 2H), 5.072 (t, *J* = 6.0 Hz, 2H), 3.964 (s, 6H); ^13^C NMR (125 MHz, CDCl_3_) δ 166.4, 158.1, 133.4, 132.4, 121.3, 121.1, 111.5, 56.3, 45.4; MS (ESI) *m/z* (%) 651.0 ([M + M + Na]^+^, 100%); IR (KBr) ν (cm^-1^) 3402.74, 2967.60, 2945.18, 1652.42, 1520.05, 1483.51, 1295.15, 1246.91, 1021.08, 759.43.

***N*,*N'*-Methylenebis(3,5-dimethoxybenzamide) (Table 2, Table 4, entry 5):**

EA/PE = 3/2 as eluant, white solid. ^1^H NMR (500 MHz, DMSO-*d*_6_) δ 8.145 (t, *J* = 6.0 Hz, 2H), 6.875 (d, *J* = 2.5 Hz, 4H), 6.430 (t, *J* = 2.0 Hz, 2H), 4.853 (t, *J* = 6.0 Hz, 2H), 3.666 (s, 12H); ^13^C NMR (125 MHz, DMSO-*d*_6_) δ 167.3, 160.5, 135.9, 105.3, 103.8, 55.4, 45.5; MS (ESI) *m/z* (%) 748.9 ([M + M]^+^, 100%); IR (KBr) ν (cm^-1^) 3304.58, 3001.10, 1600.77, 1534.61, 1207.76, 1159.24, 1107.20, 1064.11.

***N*,*N'*-Methylenebis(3-chlorobenzamide) (Table 2, Table 4, entry 7):**

EA/PE = 1/3 as eluant, white solid. ^1^H NMR (500 MHz, DMSO-*d_6_*) δ 8.514 (t, *J* = 5.5 Hz, 2H), 7.598 (t, *J* = 1.5 Hz, 2H), 7.487–7.469 (m, 2H), 7.127–7.105 (m, 2H), 7.032 (t, *J* = 7.5 Hz, 2H), 4.622 (t, *J* = 6.0 Hz, 2H); ^13^C NMR (125 MHz, DMSO-*d_6_*) δ 166.4, 136.2, 134.2, 131.6, 130.0, 128.1, 126.3, 45.8; MS (ESI) *m/z* (%) 680.7 ([M + M + Cl]^+^, 100%), 682.7 ([M + M + Cl]^+^, 44%), 683.7 ([M + M + Cl]^+^, 15%); IR (KBr) ν (cm^-1^) 3295.277, 3060.40, 1636.597, 1530.84, 1305.97, 1288.82, 1270.06, 1113.53

***N*,*N'*-Methylenebis(3-phenylacrylamide) (Table 2, Table 4, entry 9):**

EA/PE = 1/1 as eluant, white solid. ^1^H NMR (500 MHz, DMSO-*d*_6_) δ 8.806 (t, *J* = 6.0 Hz, 2H), 7.554–7.536 (m, 4H), 7.476 (d, *J* = 15.5 Hz, 2H), 7.420–7.362 (m, 6H), 6.684 (d, *J* = 15.0 Hz, 2H), 4.632 (t, *J* = 6.0 Hz, 2H); ^13^C NMR (125 MHz, DMSO-*d*_6_) δ 166.0, 140.1, 135.5, 130.3, 129.6, 128.3, 122.5, 44.2; MS (ESI) *m/z* (%) 635.0 ([M + M + Na]^+^, 100%); IR (KBr) ν (cm^-1^) 3288.59, 3059.93, 3027.03, 1653.46, 1621.39, 1538.98, 1337.43, 1213.44, 1113.30, 973.51, 767.23.

***N*,*N'*-Methylenebis(2-phenoxyacetamide) (Table 4, entry 11):**

EA/PE = 1/1 as eluant, white solid. ^1^H NMR (500 MHz, CDCl_3_) δ 7.823 (s, 1H), 7.666(s, 1H), 7.311 (t, *J* = 7.0 Hz, 4H), 7.021 (t, *J* = 7.0 Hz, 2H), 6.917 (t, *J* = 9.5 Hz, 4H), 4.858–4.844 (m, 2H), 4.508 (s, 2H), 4.474 (s, 2H); ^13^C NMR (125 MHz, CDCl_3_) δ 170.3, 169.9, 157.3, 157.2, 130.1, 130.0, 122.5, 122.4, 114.9, 114.9, 67.3, 67.3, 43.6; MS (ESI) *m/z* (%) 650.9 ([M + M + Na]^+^, 100%); IR (KBr) ν (cm^-1^) 3332.59, 3063.01, 2927.32, 1677.99, 1599.25, 1524.04, 1239.15, 1057.75, 754.55, 691.31.

***N*-(Methylthiomethyl)octanamide**

EA/PE = 1/1 as eluant, white solid. ^1^H NMR (500 MHz, CDCl_3_) δ 6.785 (s, 1H), 5.564 (d, *J* = 6.5 Hz, 2H), 2.881 (s, 3H), 2.299 (t, *J* = 7.5 Hz, 2H), 1.681–1.623 (m, 2H), 1.316–1.270 (m, 8H), 0.874 (t, *J* = 7.0 Hz, 3H); ^13^C NMR (125 MHz, CDCl_3_) δ 173.9, 59.8, 40.1, 36.4, 31.8, 29.4, 29.2, 25.6, 22.7, 14.2; MS *m/z* (%) 156.8 (17), 127.6 (74), 72.4 (6), 57.3 (100), 55.3 (20), 43.3 (43), 41.3 (38), 30.3 (33); IR (KBr) ν (cm^-1^) 3317.95, 2929.58, 1537.98, 1462.81, 1304.56, 1289.83, 1137.64, 1127.94.

# Reference

1. Ortéga, F.; Bastide, J. *Bioorg. Chem.* **1997,** *25,* 261–274. doi:[10.1006/bioo.1997.1071](http://dx.doi.org/10.1006/bioo.1997.1071)
