# Supplementary material for: Synthesis of methylenebisamides using CC- or DCMT-activated DMSO
Source: Beilstein J Org Chem. 2008 Dec 15;4:51. doi: 10.3762/bjoc.4.51 (PMC2633661; doi:10.3762/bjoc.4.51)
Supplement: File 2 — NMR spectra of new compounds [file Beilstein_J_Org_Chem-04-51-s002.doc]

# Supporting information

# Synthesis of methylenebisamides using CC- or DCMT-activated DMSO

Qiang Wang^1,2^, Lili Sun^1^, Yu Jiang^1^ and Chunbao Li^1^,*

Address: ^1^Department of Chemistry, College of Science, Tianjin University, Tianjin 300072, China and ^2^State Key Laboratory Base of Novel Functional Materials and Preparation Science at Ningbo, Faculty of Materials Science and Chemical Engineering, Ningbo University, Ningbo 315211, China.

Email: Chunbao Li* - [lichunbao@tju.edu.cn](mailto:lichunbao@tju.edu.cn)

* Corresponding author

**^1^H NMR and ^13^C NMR spectra of new compounds**
